# Association between TGF-β gene polymorphism and myopia: A systematic review and meta-analysis

**DOI:** 10.1097/MD.0000000000029961

**Published:** 2022-07-29

**Authors:** Xiaoyu Zhu, Bowei Xu, Lingxue Dai, Zuoyuan Wang, Li Feng, Jiangyue Zhao

**Affiliations:** a Department of Ophthalmology, The Fourth Affiliated Hospital of China Medical University; Shenyang 110005, China; b Eye Hospital of China Medical University; Shenyang 110005, China; c Key Lens Research Laboratory of Liaoning Province, Shenyang 110005, China; d Department of Nephrology, Huashan Hospital, Fudan University, Shanghai, 200040, China.

**Keywords:** gene polymorphism, myopia, scleral remodeling, TGF-β

## Abstract

**Introduction:**

The present study was conducted to determine the association of transforming growth factor-beta (TGF-β) gene polymorphism and myopia.

**Method:**

Four hundred twelve articles were identified, of which 11 articles with 5213 participants in 4 countries were included in the final analysis. Review Manager software (RevMan, version 5.4) was used for data analysis.

**Result:**

Odds ratio (OR) value of TGF-β1 rs1800469 is 1.33 (95% confidence interval [CI] = 1.15–1.54) in the allelic model; in the dominant model is 1.76 (95% CI = 1.16–2.67); in homozygous model is 5.98 (95% CI = 4.31–8.06). OR value of TGF-β1 rs4803455 is 0.62 (95% CI = 0.43–0.88) in recessive model. TGF-β2 is not associated with myopia. Relevant study on TGF-β3 is scarce.

**Conclusion:**

Our systematic review and meta-analysis found that TGF-β1 rs4803455 and rs1800469 were correlated with myopia.

## 1. Introduction

Myopia is a disease in which vision becomes blurred due to axial elongation of the eye and focus in front of the retina. Myopia comes on mainly with children and teenagers. In East Asia and other regions, the proportion of myopia among teenagers is increasing year by year; the highest can reach 80% to 90%, among which high myopia has accounted for >20%. Myopia has become one of the most common eye diseases in the world. The pathogenesis of myopia is still unclear, but research shows that the formation of myopia is promoted by many factors, such as environmental factors, education level, and so on. However, whether genetic factors affect the occurrence and development of myopia have been controversial.^[1–3]^

A growing number of researchers believe that there is a direct link between myopia and heredity. Ip et al Found that compared with children with no myopic parents, children with 1 myopic parent and those with 1 myopic parent had a 2-fold and 8-fold higher risk of developing myopia, respectively. In addition, increased myopia in parents also increases the risk of myopia.^[4]^ Dirani et al. found that myopia in identical twins was also correlated.^[5]^ The discovery of >20 regions of the genome associated with myopia also indicates that the origin of myopia is polygenic and heterogeneous and offers the prospect of specific targeted therapy.^[6]^

Transforming Growth Factor-Beta (TGF-β) is a family of cytokines that regulate biological development. TGFβ has evolved to regulate epithelial and neural tissue expansion systems, the immune system, and wound repair. In the field of the tumor, the TGF-β signal contributes to tumor progression, and in fibrotic diseases, TGF-β promotes the occurrence and development of pulmonary fibrosis.^[7–9]^ In recent years, experiments have shown that TGF-β also affects scleral remodeling and the formation of high myopia.^[10,11]^ The subtypes of TGF-β that can act on the sclera are mainly divided into 1, 2, and 3. At present, most human studies focus on TGF-β1, while TGF-β2,3 type studies are relatively few. Different single nucleotide polymorphisms (SNPs) in TGF-β1 have different effects on myopia. Rs1800469, rs1982073, rs2241716, and rs4803455 are the 4 SNPs that attract more attention. The results were not the same.

This article aims to present all existing researches on TGF-β polymorphisms and myopia through meta-analysis and systematic review to explore the relationship between TGF-β polymorphisms and myopia.

## 2. Methods

### 2.1. Protocol and registration

The systematic review protocol was registered at the International Prospective Register of Systematic Reviews (PROSPERO) under identification number CRD42021284441. All stages of this study were performed under PRISMA guidelines.^[1]^

### 2.2. Eligibility criteria

Inclusion criteria were as follows: there are no restrictions on the subjects’ age, race, and gender. However, studies with other complications were not included; the case-control study on the association between TGFB1 gene polymorphism and myopia; the average spherical refractive error (SRE) of the cases is less than or equal to −0.50d, and the average SRE of the controls is more excellent than −0.50d; only case-control studies were included; ff there are multiple research reports, include an extended version; and unregistered studies were not included.

Exclusion criteria were as follows: reviews, editorials, opinion papers, and other studies presenting nonoriginal data; conference papers are not included; animal study; studies with other complicating diseases; articles providing incomplete data.

### 2.3. Information sources

We searched English databases such as PubMed, EMBASE, ProQuest, PsycINFO, CINAHL, and Cochrane Library and Chinese databases such as CNKI, Sinomed, and Wanfang databases, from their establishment to October 9, 2021. The language is not limited, and the similar literature and references attached to the search results are consulted simultaneously.

### 2.4. Search strategy

We take “myopia” or “nearsightedness” or “shortsightedness” or “near‐sight” or “near‐sighted” or “near‐sightedness” or “short‐sight” or “short‐sighted” or “short‐sightedness” or “refractive error” and “Transforming Growth Factor beta” or “Milk Growth Factor” or “Factor, Milk Growth” or “Growth Factor, Milk” or “TGF-beta” or “TGFbeta” or “Platelet Transforming Growth Factor” or “Bone-Derived Transforming Growth Factor” or “Bone Derived Transforming Growth Factor” or “TGFB” and “Polymorphism, Single Nucleotide” or “Nucleotide Polymorphism, Single” or “Nucleotide Polymorphisms, Single” or “Polymorphisms, Single Nucleotide” or “Single Nucleotide Polymorphisms” or “SNP” or “SNPs” or “Single Nucleotide Polymorphism” as English keywords, and “Jinshi” or “Quguangbuzheng” or “Quguang” and “Zhuanhuashengzhangyinziβ” or “Zhuanhuashengzhangyinzibeta” or “TGFbeta” or “TGFβ” or “TGFB” and “Yiganjiyinduotaixing” or “Danhegansuanduotaixing” or “Jiyinduotaixing” or “SNP” as Chinese keywords, and use these to formulate retrieval strategies. The specific retrieval strategy is shown in the appendix.

### 2.5. Data extraction process

Two authors independently screened all search results. The 2 authors independently extracted the data in the study using a precustomized data table. The data sheet extracted the information of the first author, the year of publication, the polymorphism of the study, the total sample size, genotype frequency, allele frequency, age, race, the definition of the case group and the control group, whether each study met the Hardy-Weinberg balance, and whether the polymorphism in the experiment was associated with myopia. First, randomly select ten publications, and 2 reviewers perform calibration exercises to clarify whether the data extraction method and the variables extracted by the data extraction form need improvement. After 2 people extract data independently, they will consult where they disagree. If 2 authors cannot reach an agreement, ask another author to intervene. No automated tools were used except Noteexpress for duplicate checking. If there is no allele or genotype data in the report and whether the Hardy-Weinberg equilibrium is reached, we will calculate it ourselves.

### 2.6. Quality assessment

We used CASP Systematic Review Checklist^[2]^ to evaluate the included studies. Evaluation criteria include:

1. Was there a clear statement of the aims of the research?
2. Is a qualitative methodology appropriate?
3. Was the research design appropriate to address the aims of the research?
4. Was the recruitment strategy appropriate to the aims of the research?
5. Was the data collected in a way that addressed the research issue?
6. Has the relationship between researcher and participants been adequately considered?
7. Have ethical issues been taken into consideration?
8. Was the data analysis sufficiently rigorous?
9. Is there a clear statement of findings?
10. How valuable is the research?

### 2.7. Statistical analysis

The meta-analysis was performed if the SNP had at least 2 study evaluations. Dominant, recessive, homozygous, heterozygous, and allele models were used to analyze the correlation between SNP and myopia. We used Review Manager software (RevMan, version 5.4) for data analysis. The fixed effect and random effect models were combined with odds ratio (OR) and 95% confidence interval (95% CI) to evaluate the association strength between SNP and myopia in the combined sample. The Cochrane I^2^ tested the statistical heterogeneity of the included studies. The fixed effect model is used if *P* > .1, I^2^ ≤ 50%, indicating low heterogeneity, If *P* < .1, I^2^ > 50%, indicating high heterogeneity, the random effect model is used.^[12]^ One-way sensitivity analysis was performed by sequentially excluding each study in the meta-analysis. The possibility of publication bias was assessed by visual inspection of the funnel chart. The standard error of each study’s logarithm (OR) was plotted with its corresponding logarithm (OR). If there is an asymmetric chart, it indicates that there may be publication bias.

## 3. Results

### 3.1. Search results

**Figure 1.**
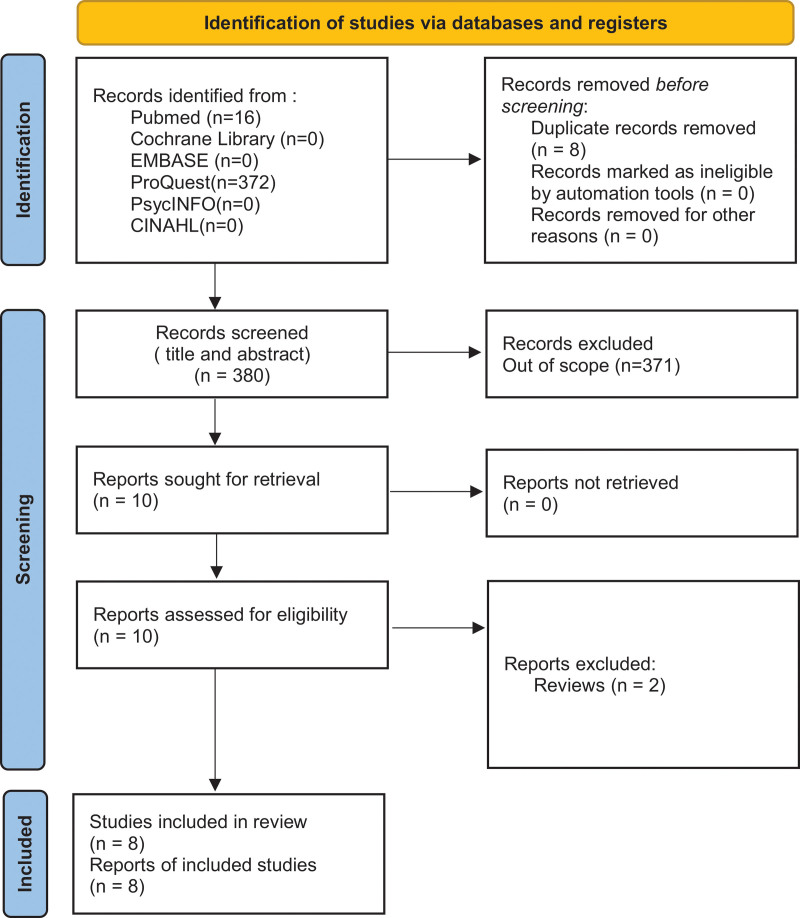
PRISMA Flow Diagram of English article screening process.

**Figure 2.**
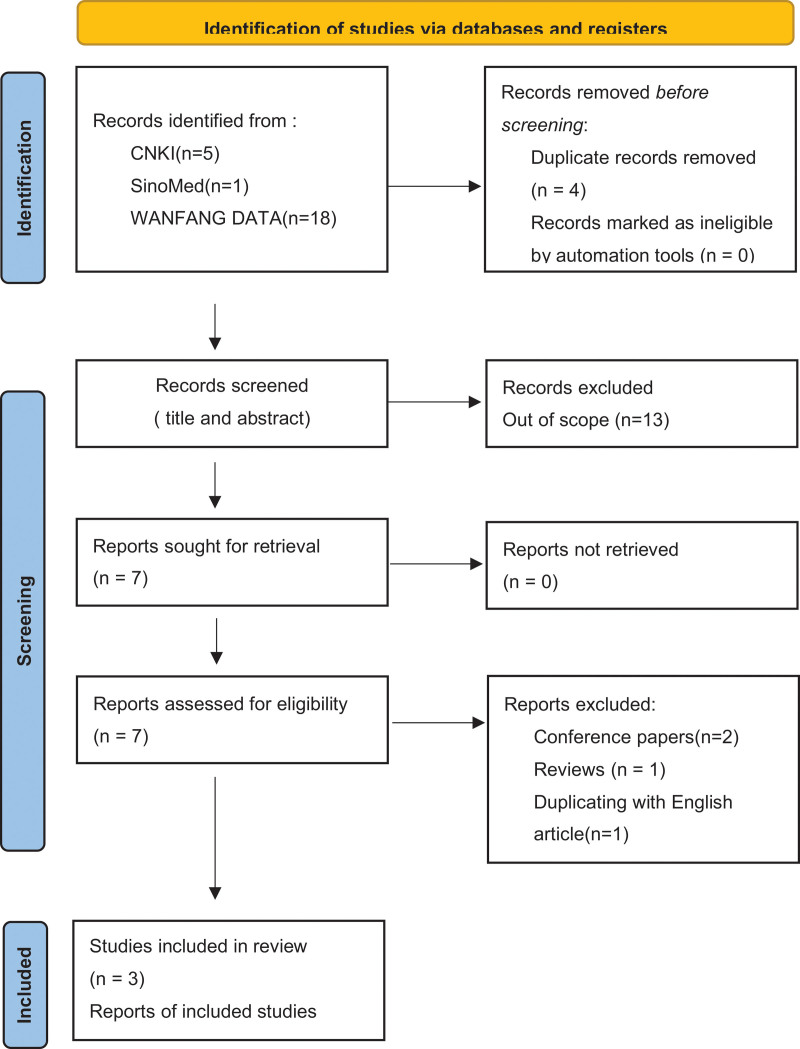
PRISMA Flow Diagram of Chinese article screening process.

The article screening process is shown in Figures 1 and 2. Four hundred twelve studies were retrieved, including 388 in English and 24 in Chinese. Finally, 11 studies were included in the meta-analysis, including 8 English and 3 Chinese studies. The 11 characteristics are shown in Tables 1–3. Nine studies were TGFbeta1 related. Nine studies were TGFbeta2 related. There was only 1 SNP study of TGFbeta3, which could not be included in the meta-analysis. In the included study, there were 2981 patients in the case group and 2232 patients in the control group.

**Table 1:** Basic characteristics of included studies on SNP of TGFbeta1.

First author	Year	Race	SNP ID	Sample size		Age(year)		Definition of cases (SRE) (D)	Definition of controls (SRE) (D)	HWE	Whether associated to myopia	Quality score
				Cases	Controls	Cases	Controls					
Rasool	2013	Kashmiri	Rs1982073	247	176	Cannot tell		≤−6.00	Cannot tell	Yes	Yes	8
			Rs1800471							Yes	No	
			Novel variant							Yes	No	
Lin	2006	Chinese	Rs1982073	201	86	16–25		≤−6.00	≥−0.50	Yes	Yes	8
Hayashi	2007	Japanese	Rs1800820	330	330	37.82 ± 11.97	Cannot tell	≤−9.25	≥−2.00	Cannot tell	No	8
			Rs1054797								No	
			Rs1800468								No	
			Rs1800469								No	
			Rs2241715								No	
			Rs11466324								No	
			Rs2241717								No	
			Rs11672143								No	
			Rs11466334								No	
			Rs2278422								No	
Wang	2009	Chinese	Rs1982073	288	208	21.76 ± 16.24	27.32 ± 7.32	≤−6.00	−0.50 to +1.00	Yes	No	9
			Rs2229336							Yes	No	
Zha	2009	Chinese	Rs1800469	300	300	15–48	17–46	−24.00 to −8.00	−1.00 to +0.88	Yes	Yes	9
			Rs1800470							Yes	Yes	
			Rs2241716							Yes	Yes	
			Rs4803455							No	Yes	
			Rs11466345							Yes	No	
			Rs12983047							Yes	No	
			Rs10417924							Yes	No	
			Rs12981053							Yes	No	
Khor	2010	Chinese	Rs4803455	630	348	10–12		≤−0.50	≥−0.50	Yes	No	8
Zha	2008	Chinese	Rs1982073	300	300	Cannot tell		≤−8.00	±0.75	Yes	Yes	8
Shi	2017	Chinese	Rs1800469	73	103	12–18 (14 ± 1.58)		≤−0.50	>−0.50	Yes	No	8
			Rs2241716	67	103					Yes	Yes	
			Rs4803455	66	103					Yes	No	
Liu	2019	Chinese	Rs4803455	343	210	10.58 ± 2.42	8.62 ± 2.12	≤−0.50	>−0.50	No	Cannot tell	7
			Rs2241716							Yes		
			Rs1800469							No		
Biler	2018	Turkish	Rs4803455	74	77	7.1 ± 3	9.6 ± 1.8	≤−6.00D	≥−0.50D	Yes	No	7

**Table 2:** Basic characteristics of included studies on SNP of TGFbeta2.

First author	Year	Race	SNP ID	Sample size		Age (year)		Definition of cases (SRE) (D)	Definition of controls (SRE) (D)	HWE	Whether associated to myopia	Quality score
				Cases	Controls	Cases	Controls					
Lin	2009	Chinese	Rs7550232	195	94	17–24	17–25	≤−6.50	−0.50 to +1.00	Yes	Yes	9
			Rs991967							Yes	No	
Shi	2017	Chinese	Rs1473527	67	103	12–18 (14 ± 1.58)		≤−0.50	>−0.50	Yes	No	8
			Rs6604604							Yes	No	
			Rs6691070							Yes	No	
			Rs7750232							Yes	No	
			Rs900							Yes	No	

**Table 3:** Basic characteristics of included studies on SNP of TGFbeta3.

First author	Year	Race	SNP ID	Sample size		Age (year)		Definition of cases (SRE)(D)	Definition of controls (SRE)(D)	HWE	Whether associated to myopia	Quality score
				Cases	Controls	Cases	Controls					
Zha	2008	Chinese	Rs2268626	300	300	Cannot tell		≤−8.00	±0.75	Yes	No	8
			Rs3917158							Yes	No	
			Rs4252328							Yes	No	
			Rs3917192							Yes	No	
			Rs3917201							Yes	No	
			Rs3917205							Yes	No	
			Rs2284791							Yes	No	

Four SNPs of TGFbeta1 were finally investigated by more than 2 studies; they were rs1982073, rs2241716, rs1800469, rs4803455. Only rs7550232 of TGFbeta2 was investigated by 2 studies. The meta-analyses under the 5 genetic models are shown in Tables 4 and 5.

**Table 4:** Results of TGFβ1 SNP meta-analysis.

SNPs	Genetic models	Number of studies	Events		Pooled OR (95% CI)		*P*		Heterogeneity		
			Cases	Controls	FEM	REM	FEM	REM	Q	P_Q_	I^2^ (%)
rs1982073	C vs T	4	1118/2070	736/1540	1.31 [1.15, 1.50]	1.36 [1.01, 1.84]	<.0001	.05	14.21	0.003	79
	CC+CT vs TT	4	819/1035	546/770	1.56 [1.26, 1.95]	1.64 [1.04, 2.58]	<.0001	.03	12.07	0.007	75
	CT vs TT	4	520/736	356/580	1.54 [1.02, 2.33]	1.48 [1.17, 1.86]	.001	.03	8.90	0.03	66
	CC vs TT	4	299/515	190/414	1.71 [1.30, 2.24]	1.90 [1.01, 3.55]	.0001	.05	14.11	0.003	79
	CC vs CT+TT	4	299/1035	190/770	1.31 [1.05, 1.63]	1.39 [0.92, 2.09]	.01	.12	9.15	0.03	67
rs1800469	A vs G	4	974/1602	750/1414	1.33 [1.15, 1.54]	1.33 [1.15, 1.54]	.0001	.0001	0.94	0.82	0
	AA+AG vs GG	3	621/706	493/613	1.70 [1.25, 2.32]	1.76 [1.16, 2.67]	.007	.008	3.21	0.20	38
	AG vs GG	3	361/446	323/443	1.67 [0.97, 2.89]	1.67 [0.97, 2.89]	.009	.07	4.90	0.09	59
	AA vs GG	3	260/342	170/493	5.89 [4.31, 8.06]	5.89 [4.31, 8.06]	<.00001	<.00001	0.42	0.81	0
	AA vs AG+GG	3	260/706	170/613	1.46 [1.15, 1.85]	1.46 [1.15, 1.85]	.002	.002	1.50	0.47	0
rs4803455	A vs C	5	955/2826	780/2076	0.85 [0.76, 0.96]	0.85 [0.69, 1.03]	.01	.10	9.36	0.05	57
	AA+AC vs CC	5	818/1413	623/1038	0.93 [0.79, 1.09]	0.89 [0.67, 1.18]	.37	.41	9.54	0.05	58
	AC vs CC	5	681/1276	466/881	1.03 [0.87, 1.23]	0.98 [0.75, 1.29]	.72	.90	8.28	0.08	52
	AA vs CC	5	137/732	157/572	0.61 [0.47, 0.80]	0.61 [0.39, 0.95]	.0003	.03	9.07	0.06	56
	AA vs AC+CC	5	137/1413	157/1038	0.60 [0.47, 0.77]	0.62 [0.43, 0.88]	<.0001	.008	6.97	0.14	43
rs2241716	T vs C	3	374/1420	387/1226	0.74 [0.63, 0.88]	0.53 [0.24, 1.15]	.0008	.11	31.13	<0.00001	94
	TT+TC vs CC	3	326/710	329/613	0.69 [0.55, 0.86]	0.45 [0.17, 1.19]	.0008	.11	30.08	<0.00001	93
	TC vs CC	3	278/662	271/455	0.45 [0.35, 0.57]	0.26 [0.06, 1.12]	<.00001	.07	47.23	<0.00001	96
	TT vs CC	3	48/432	58/342	0.60 [0.40, 0.91]	0.50 [0.15, 1.64]	.02	.25	12.29	0.0002	84
	TT vs TC+CC	3	48/710	58/613	0.69 [0.46, 1.05]	0.63 [0.25, 1.61]	.08	.34	7.91	0.02	75

**Table 5:** Results of TGFβ2 SNP meta-analysis.

SNPs	Genetic models	Number of studies	Events	Pooled OR (95% CI)	*P*	Heterogeneity	FEM	REM	Q	P_Q_	I^2^ (%)
			Cases	Controls	FEM	REM					
rs7550232	T vs C	2	282/322	537/596	0.76 [0.50, 1.16]	0.94 [0.26, 3.37]	0.20	0.92	6.81	0.009	85
	TT+TC vs CC	2	160/161	294/298	1.69 [0.27, 10.66]	1.55 [0.23, 10.30]	0.58	0.65	0.40	0.53	0
	TC vs CC	2	38/39	51/55	2.00 [0.29, 13.75]	1.99 [0.29, 13.72]	0.48	0.49	0.01	0.91	0
	TT vs CC	2	122/123	243/247	1.59 [0.25, 9.92]	1.41 [0.21, 9.38]	0.82	0.72	0.59	0.44	0
	TT vs TC+CC	2	122/161	243/298	0.69 [0.44, 1.10]	0.85 [0.22, 3.31]	0.12	0.82	6.72	0.01	85

### 3.2. Association between TGFbeta1 rs1982073 and myopia

**Figure 3.**
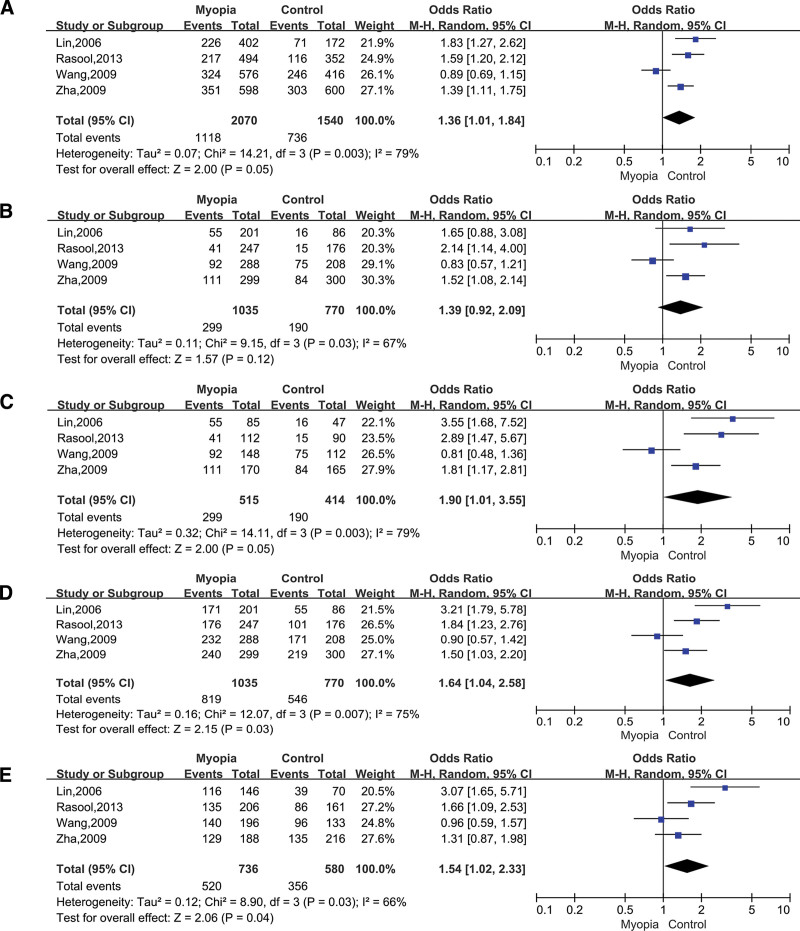
Forest plots of the pooled ORs with 95% CIs for associations between TGFbeta1 rs1982073 and myopia. The bars with squares in the middle represent 95% CIs and ORs. The central vertical solid line indicates the ORs for the null hypothesis. Diamond indicates summary OR with its corresponding 95% CI. (A) Allelic model (C vs T); (B) Dominant model (CC+CT vs TT); (C) Heterozygous model (CT vs TT); (D) Homozygous model (CC vs TT); (E) Recessive model (CC vs CT+TT). CIs = confidence intervals, ORs = odds ratios.

Four studies were about rs1982073, which included 1035 cases and 770 controls. The result of 5 model shows all of 95% CI >1, *P* value <.1, I^2^ > 50%, which showed that TGFbeta1 rs1982073 had no association with myopia. Allelic model (C vs T; OR = 1.36; 95% CI = 1.01–1.84; *P* = .05 in the random-effects model; Fig. 3A), Dominant model (CC+CT vs TT; OR = 1.64; 95% CI = 1.04−2.58; *P* = .03 in the random-effects model; Fig. 3B), Heterozygous model (CT vs TT; OR = 1.54; 95% CI = 1.02−2.33; *P* = .03 in the random-effects model; Fig. 3C), Homozygous model (CC vs TT; OR = 1.90; 95% CI = 1.01−3.55; *P* = .05 in the random-effects model; Fig. 3D), and Recessive model (CC vs CT+TT; OR = 1.31; 95% CI = 1.05–1.63; *P* = .01 in the random-effects model; Fig. 3E).

### 3.3. Association between TGFbeta1 rs1800469 and myopia

**Figure 4.**
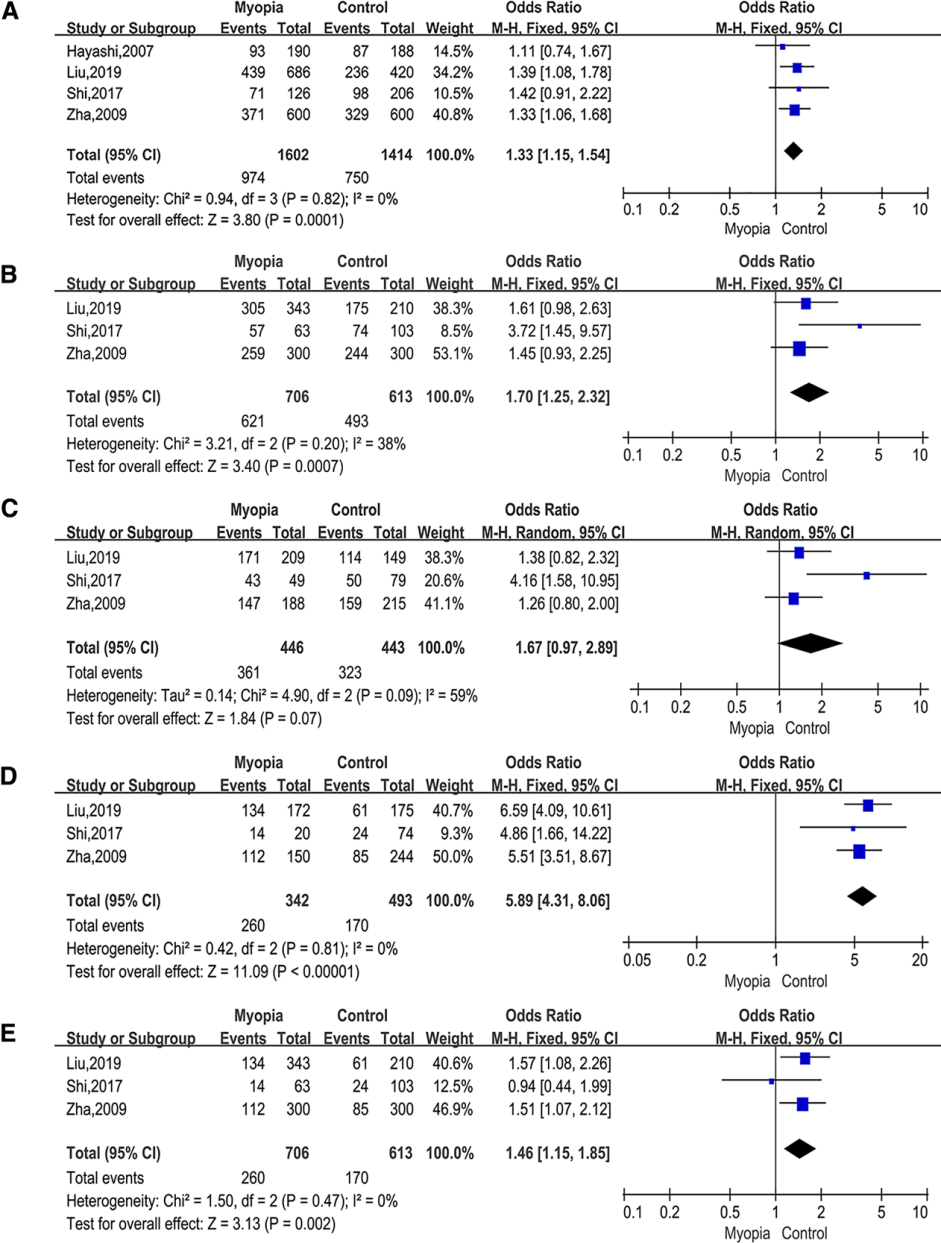
Forest plots of the pooled ORs with 95% CIs for associations between TGFbeta1 rs1800469 and myopia. The bars with squares in the middle represent 95% CIs and ORs. The central vertical solid line indicates the ORs for the null hypothesis. Diamond indicates summary OR with its corresponding 95% CI. (A) Allelic model (A vs G); (B) Dominant model (AA+AG vs GG); (C) Heterozygous model (AG vs GG); (D) Homozygous model (AA vs GG); (E) Recessive model (AA vs AG+GG). CIs = confidence intervals, ORs = odds ratios.

A total of 4 studies were about rs1800469, which included 801 cases and 707 controls. Our data displayed allelic model (A vs G; OR = 1.33; 95% CI = 1.15–1.54; *P* = .0001 in the fixed-effects model; Fig. 4A), dominant model (AA+AG vs GG; OR = 1.76; 95% CI = 1.16–2.67; *P* = .008 in the fixed-effects model; Fig. 4B), heterozygous model (AG vs GG; OR = 1.67; 95% CI = 0.97–2.89; *P* = .07 in the random-effects model; Fig. 4C), homozygous model (AA vs GG; OR = 5.98; 95% CI = 4.31–8.06; *P* < .00001 in the fixed-effects model; Fig. 4D), and recessive model (AA vs AG+GG; OR = 1.46; 95% CI = 1..15–1..85; *P* = .002 in the fixed-effects model; Fig. 4E). The models showed that TGFbeta1 rs1800469 had a significant association with myopia besides the heterozygous model.

### 3.4. Association between TGFbeta1 rs4803455 and myopia

**Figure 5.**
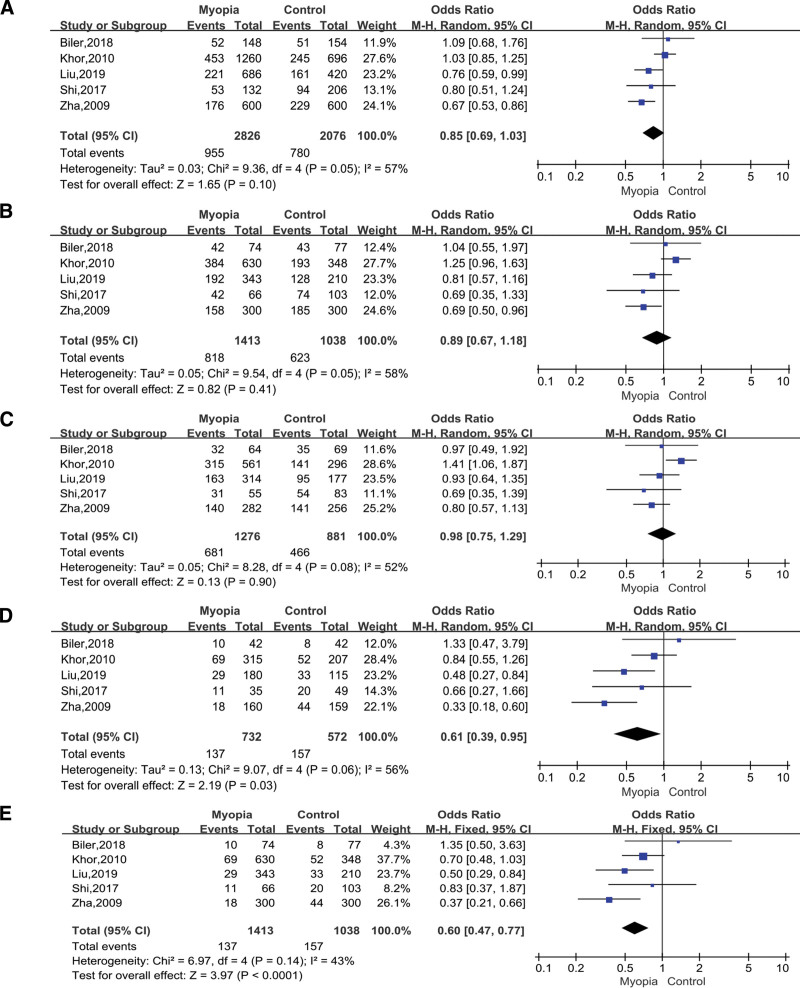
Forest plots of the pooled ORs with 95% CIs for associations between TGFbeta1 rs4803455 and myopia. The bars with squares in the middle represent 95% CIs and ORs. The central vertical solid line indicates the ORs for the null hypothesis. Diamond indicates summary OR with its corresponding 95% CI. (A) Allelic model (A vs C); (B) Dominant model (AA+AC vs CC); (C) Heterozygous model (AC vs CC); (D) Homozygous model (AA vs CC); (E) Recessive model (AA vs AC+CC). CIs = confidence intervals, ORs = odds ratios.

A total of 5 studies were about rs4803455, which included 1913 cases and 1038 controls. We listed the allelic model (A vs C; OR = 0.85; 95% CI = 0.69–1.03; *P* = .10 in the random-effects model; Fig. 5A), dominant model (AA+AC vs CC; OR = 0.89; 95% CI = 0.67–1.18; *P* = .41 in the random-effects model; Fig. 5B), heterozygous model (AC vs CC; OR = 1.03; 95% CI = 0.75–1.29; *P* = .90 in the random-effects model; Fig. 5C), homozygous model (AA vs CC; OR = 0.61; 95% CI = 0.39–0.95; *P* = .03 in the random-effects model; Fig. 5D), and recessive model (AA vs AC+CC; OR = 0.62; 95% CI = 0.43–0.88; *P* < .0001 in the fixed-effects model; Fig. 5E). The only recessive model showed that TGFbeta1 rs4803455 had an association with myopia.

### 3.5. Association between TGFbeta1 rs2241716 and myopia

**Figure 6.**
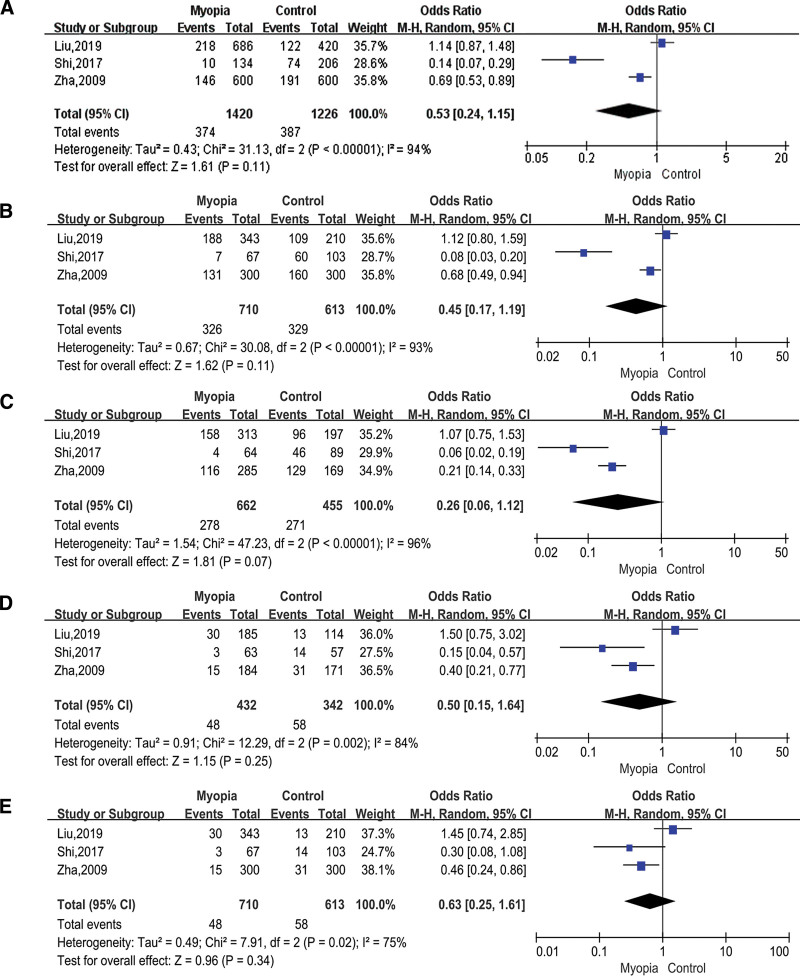
Forest plots of the pooled ORs with 95% CIs for associations between TGFbeta1 rs2241716 and myopia. The bars with squares in the middle represent 95% CIs and ORs. The central vertical solid line indicates the ORs for the null hypothesis. Diamond indicates summary OR with its corresponding 95% CI. (A) Allelic model (T vs C); (B) Dominant model (TT+TC vs CC); (C) Heterozygous model (TC vs CC); (D) Homozygous model (TT vs CC); (E) Recessive model (TT vs TC+CC). CIs = confidence intervals, ORs = odds ratios.

A total of 3 studies were about rs2241716, which included 710 cases and 613 controls. Allelic model (T vs C; OR = 0.53; 95% CI = 0.24–1.15; *P* = .11 in the random-effects model; Fig. 6A), dominant model (TT+TC vs CC; OR = 0.45; 95% CI = 0.17–1.19; *P* = .11 in the random-effects model; Fig. 6B), heterozygous model (TC vs CC; OR = 0.26; 95% CI = 0.06–1.12; *P* = .07 in the random-effects model; Fig. 6C), homozygous model (TT vs CC; OR = 0.50; 95% CI = 0.15–1.54; *P* = .25 in the random-effects model; Fig. 6D), and recessive model (TT vs TC+CC; OR = 0.69; 95% CI = 0.46–1.05; *P* = .08 in the random-effects model; Fig. 6E), all showed that TGFbeta1 rs2241716 had no association with myopia.

### 3.6. Association between TGFbeta2 rs7550232 and myopia

**Figure 7.**
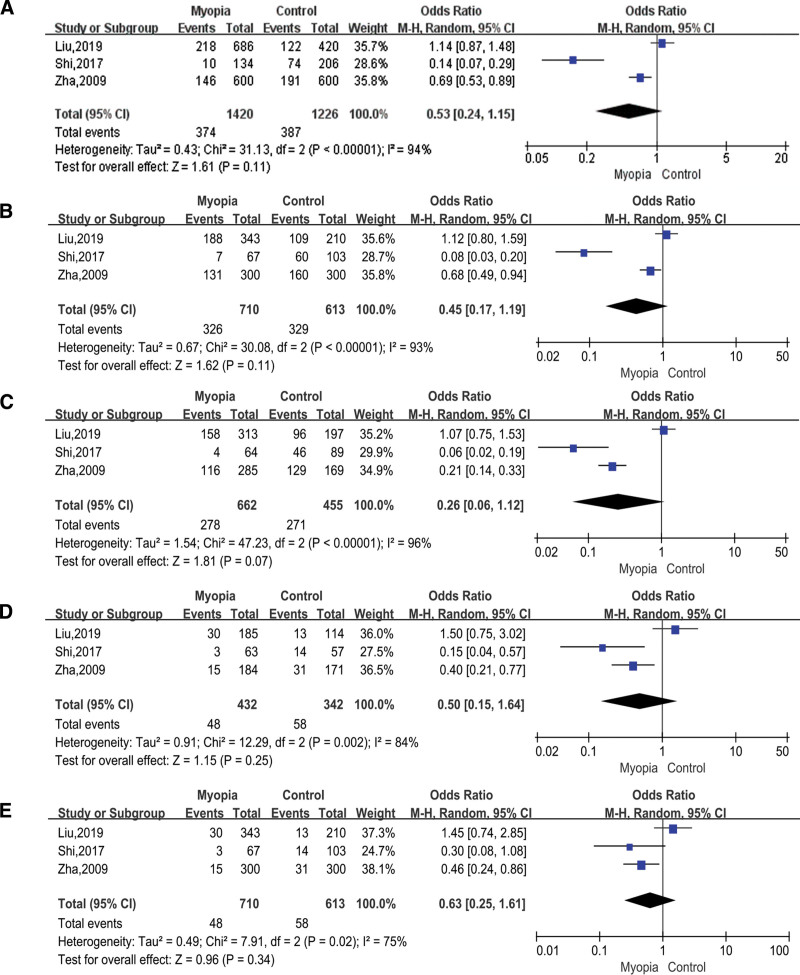
Forest plots of the pooled ORs with 95% CIs for associations between TGFbeta2 rs7550232 and myopia. The bars with squares in the middle represent 95% CIs and ORs. The central vertical solid line indicates the ORs for the null hypothesis. Diamond indicates summary OR with its corresponding 95% CI. (A) Allelic model (T vs C); (B) Dominant model (TT+TC vs CC); (C) Heterozygous model (TC vs CC); (D) Homozygous model (TT vs CC); (E) Recessive model (TT vs TC+CC). CIs = confidence intervals, ORs = odds ratios.

A total of 2 studies were about rs7550232, which included 161 cases and 298 controls. Allelic model (T vs C; OR = 0.94; 95% CI = 0.26–3.37; *P* = .92 in the random-effects model; Fig. 7A), dominant model (TT+TC vs CC; OR = 1.69; 95% CI = 0.27–10.66; *P* = .11 in the fixed-effects model; Fig. 7B), heterozygous model (TC vs CC; OR = 1.99; 95% CI = 0.29–13.72; *P* = .49 in the fixed-effects model; Fig. 7C), homozygous model (TT vs CC; OR = 1.59; 95% CI = 0.25–9.92; *P* = .62 in the fixed-effects model; Fig. 7D), and recessive model (TT vs TC+CC; OR = 0.69; 95% CI = 0.44–1.10; *P* = .12 in the random-effects model; Fig. 7E), all showed that TGFbeta2 rs7550232 had no association with myopia.

### 3.7. Sensitivity analysis

**Figure 8.**
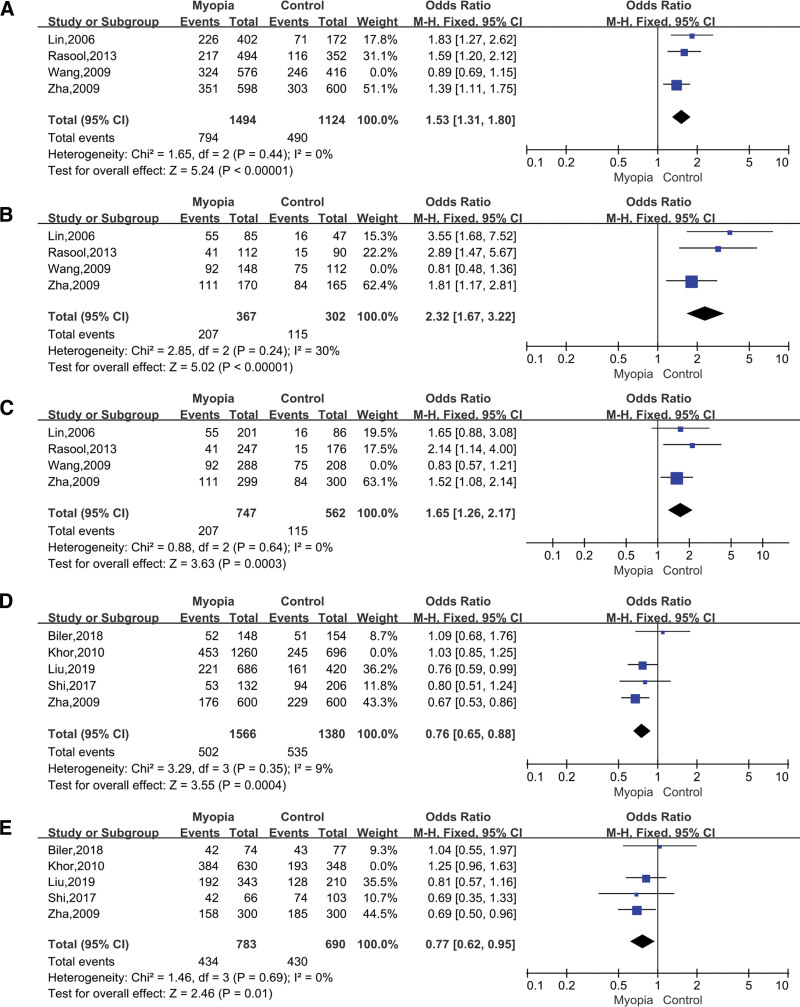
Forest plots of the pooled ORs with 95% CIs for associations between TGFbeta1 rs1982073 (A) Allelic model (C vs T); (B) Homozygous model (CC vs TT); (C) Recessive model (CC vs CT+TT) and rs4803455 (D) Allelic model (A vs C); (E) Dominant model (AA+AC vs CC)with myopia after sensitivity analysis. The bars with squares in the middle represent 95% CIs and ORs. The central vertical solid line indicates the ORs for the null hypothesis. Diamond indicates summary OR with its corresponding 95% CI. CIs = confidence intervals, ORs = odds ratios.

We performed sensitivity analyses for each genetic model for each SNP. After removing Wang et al’s study in rs1982073, heterogeneity of allelic model (C vs T; OR = 1.53; 95% CI = 1.31–1.80; *P* < .00001 in the fixed-effects model; Fig. 8A) homozygous model (CC vs TT; OR = 2.32; 95% CI = 1.67–3.22; *P* < .00001 in the fixed-effects model; Fig. 8B), recessive model (CC vs CT+TT; OR = 1.65; 95% CI = 1.26–2.17; *P* = .0003 in the fixed-effects model; Fig. 8C) was significantly reduced and overturned the previous conclusion, which showed that TGFbeta1 rs1982073 was significantly associated with myopia. After removing Khor et al’s study in rs4803455, Allelic model (A vs C; OR = 0.76; 95% CI = 0.65–0.88; *P* = .0004 in the fixed-effects model; Fig. 8D) and Dominant model (AA+AC vs CC; OR = 0.77; 95% CI = 0.62–0.95; *P* = .01 in the fixed-effects model; Fig. 8D) indicated the association of rs4803455 with myopia.

Since less than 9 studies were included in each meta-analysis, we did not assess publication bias.

## 4. Discussion

A major cause of myopia is that the sclera structure produces significant changes in tissue loss. McBrien et al found that the response of scleral thinning and scleral tissue loss was consistent in time. The reduction in collagen accumulation was most significant in the early stages of myopia development.^[13,14]^ Siegwart et al further confirmed that the scleral dry weight loss in myopia development is mainly the result of decreased collagen accumulation.^[15,16]^ Gentle et al’s study showed that collagen synthesis was reduced at the early stage of myopia development. After labeling, the degree of collagen elimination was consistent with the change of scleral dry weight previously reported.^[17,18]^ Therefore, it can be concluded that the decrease in scleral collagen accumulation during myopia is due to a reduction in collagen synthesis and an increase in collagen degradation.

TGF-β plays an essential role in many aspects of ophthalmology by regulating extracellular matrix turnover. These include nearsightedness, the development of retinal fibrosis, corneal epithelial regeneration, and blemishes after LASIK.^[19–21]^ Jobling et al reported in experiments that 3 TGF-β were found in scleral tissue to control fibroblasts and thus regulate collagen production.^[20]^ As myopia began to progress, levels of all 3 types of TGF-β decreased. The magnitude of the decrease was similar to the reduction of scleral collagen synthesis and increase in collagen breakdown in myopia.^[17]^ This may also be one of the principles of the TGF-β pathway affecting scleral remodeling to regulate the development of myopia. In addition, TGF-β also reduces glycosaminoglycan synthesis and changes integrin expression.^[22]^ Combined with McBrien et al’s study on the timeliness of scleral changes, we believe that TGF-β modifications may not be limited to studies on high myopia. In addition to affecting the development and metabolism of the sclera, TGF-β can also directly affect the outcome of myopia by influencing the differentiation of fibroblast into myofibroblast.^[23]^

Meng et al sorted out the previous studies on TGF-β1 and high myopia in 2015 and published a meta-analysis on the correlation between TGF-β1 and high myopia. On this basis, we added the studies on TGF-β and myopia published in recent years.^[24]^

Rs1982073 is located in introns, and we included 4 pieces of literature related to Rs1982073, 3 of which suggested that Rs1982073 was associated with myopia.^[11,25,26]^ However, Wang et al’s study did not prove that Rs1982073 was related to high myopia.^[27]^ Rasool et al included Kashmiri of India; Lin et al had Chinese people living in a different area of Taiwan. Zha et al took the southern Chinese, and Wang et al only stated that Chinese were included but did not specify the region. The nationalities of Chinese subjects were not identified in the 3 studies. In our result, divide by. Except for the Recessive model (CC vs CT+TT), the OR value range of Rs1982073 is >1, which is consistent with Meng et al. However, it is different from Meng’s conclusion that Rs1982073 is closely related to myopia, the results of the meta-analysis show that the heterogeneity of the included studies is high (I^2^ > 50%, *P* < .1), so the conclusion can not prove the correlation. Therefore, we are relatively conservative and believe that in the absence of clear evidence, Rs1982073 was not associated with high myopia. Sensitivity analysis was made for Rs1982073, and the exclusion of Wang et al’s study significantly reduced heterogeneity in the allelic model (C vs T), homozygous model (CC vs TT), and recessive model (CC vs CT+TT). We considered that the source of heterogeneity might be caused by different inclusion and exclusion criteria of selected subjects, which had nothing to do with race.

In the study of Rs1800469, all models except the heterozygous model had good heterogeneity, and the 95% CI was more significant than 1. We regarded that Rs1800469 was directly related to myopia development, and allele A has a protective effect on myopia. Rs1800469 was the SNP with the highest positive result in this meta-analysis, and subsequent experiments focusing on Rs1800469 might obtain better results. Among the 4 groups of positive models, the allelic model included Hayashi et al’s study and other models. Only Hayashi et al’s experiment believed that Rs1800469 at the allelic model level had nothing to do with myopia.^[28]^

As can be seen from the results, Rs4803455 showed a correlation in the recessive model (TT vs TG+GG), and it can be believed that allele T may promote myopia. Among the 5 included studies, Shi et al found that Rs4803455 was correlated with myopia in 4 Chinese studies^[10,26,29,30]^, but Biler et al found no association between Rs4803455 and myopia in the study of Turkish subjects.^[31]^ The sensitivity analysis, excluding Biler et al’s investigation, did not affect the results, indicating that race does not have a significant influence on the study. However, when the survey of Khor et al, was excluded, the heterogeneity of all models except the recessive model was significantly reduced. At that time, the allelic model and dominant model produced positive results, indicating that allele T was also a risk factor for myopia after excluding the study of Khor et al, which is consistent with the previous results of the recessive model. So, we estimated Rs4803455 was correlated with myopia.

All 3 trials involving Rs2241716 involved Chinese subjects, and the authors all considered Rs2241716 was associated with myopia.^[26,29,30]^ However, after meta-analysis, we found that all model analyses of Rs2241716 had no positive results, and the sensitivity analysis had no change. Therefore, we believe that Rs2241716 has nothing to do with myopia.

Up to now, there are few studies on TGF-β2 and TGF-β3. We retrieved 2 pieces of literature on the analysis of TGF-β2 SNP RS7550232. Shi et al reported that TGF-β2 was not associated with myopia, while Lin et al. estimated TGF-β2 is associated with high myopia.^[29,32]^ Meta-analysis showed that RS7550232 was not associated with myopia. Only Zha et al reported a study on TGF-β3 and found that TGF-β3 did not affect high myopia.^[33]^ Therefore, we believe that the current study cannot prove that TGF-β2 and TGF-β3 have an impact on the occurrence and development of myopia, and we expect that more relevant studies will be reported in the future, and new conclusions may be made.

### 4.1. Limitation

1. The retrieval scheme of this meta is the study on TGF-β and myopia. However, a meta-analysis could not be conducted due to the small literature related to TGF-β3 and the cumulative number of SNPs involved in the study being <2. Therefore, the retrieval of this meta-analysis centered on TGF-β, but the main conclusions were centered on TGF-β1 and TGF-β2.
2. The retrieval object of this study is myopia. However, most studies of TGF-β still focus on high myopia, so the results of SNP Rs1982073 can only prove that there is no correlation with high myopia but not with myopia.
3. The search scope is mainly English and Chinese databases, and the languages are English and Chinese. Other languages are not included, so there may be omissions.

## 5. Conclusion

Through the above studies, it can be concluded that Rs1800469 and Rs4803455 in TGF-β1 are associated with myopia, suggesting that further research and treatment of myopia on this basis may be of great significance. Rs1982073, Rs2241716 of TGF-β1, and Rs7550232 of TGF-β2 were not significantly associated with intolerance. TGF-β3 needs to be further studied.

## Acknowledgments

Thanks to Professor Jing Zhang (George Institute for Global Health, China) and Ziyan Yu (The Fourth Affiliated Hospital of China Medical University) for their helps with this article. Thanks to all reviewers for their valuable comments.

## Author contributions

Xiaoyu Zhu:Selected direction,Data statistics,Writing article;

Bowei Xu;Selected direction,Literature retrieval

Lingxue Dai;Writing article;

Zuoyuan Wang:Data statistics,

Li Feng;Literature retrieval

Jiangyue Zhao*:Selected direction.
